# Severity of activity limitation and prevalence of long COVID: a nationally representative cross-sectional study of adults in Scotland

**DOI:** 10.1186/s41182-026-01091-6

**Published:** 2026-09-28

**Authors:** Yusuff Adebayo Adebisi, Mubarak S. Aldosari, Yasir Ayed Alsamiri, Haroon N. Alsager

**Affiliations:** 1 Global Health Focus, Kigali, Rwanda; 2 College of Social Sciences, University of Glasgow, Glasgow, Scotland, UK; 3 Department of Clinical and Experimental Medicine, University of Catania, Catania, Italy; 4 Department of Special Education, Prince Sattam Bin Abdulaziz University, Alkharj, Saudi Arabia; 5 Department of Education, Islamic University of Madinah, Madinah, Saudi Arabia; 6 College of Science and Humanities, Prince Sattam Bin Abdulaziz University, Al-Kharj, Saudi Arabia

**Keywords:** Long COVID, Post-acute COVID-19 syndrome, Activity limitation, Disability, Health inequities, Population surveillance, Cross-sectional studies, Scotland

## Abstract

**Background:**

Long COVID is a persistent contributor to population morbidity, and people with disabilities experience substantial and often avoidable health disadvantage worldwide. Whether long COVID is disproportionately reported among adults with current functional limitations is, therefore, a question of health equity, yet nationally representative evidence from the post-emergency phase is limited. We examined the association between severity of current activity limitation and self-reported long COVID among community-dwelling adults in Scotland.

**Methods:**

We pooled cross-sectional data from the nationally representative 2023 and 2024 Scottish Health Surveys. Participants were adults aged 16 years or older with complete data on activity limitation, long-COVID status, and covariates (*N* = 9,312). Activity limitation was classified as none, limited a little, or limited a lot. Survey-weighted logistic regression accounted for calibration weights, stratification, and clustering, and adjusted for age, sex, ethnicity, education, area deprivation, cigarette-smoking status, and survey year. Average marginal predictions estimated adjusted probabilities and absolute differences.

**Results:**

Survey-weighted long-COVID prevalence was 7.59% (95% CI 6.91–8.34) overall, rising from 4.89% (95% CI 4.21–5.67) among adults without activity limitation to 9.23% (95% CI 7.70–11.04) among those limited a little and 14.42% (95% CI 12.57–16.49) among those limited a lot. Fully adjusted odds ratios were 2.07 (95% CI 1.61–2.65) and 3.26 (95% CI 2.56–4.15), respectively, versus no limitation. The adjusted absolute difference for limited a lot was 9.22 percentage points (95% CI 7.02–11.42), about nine additional cases per 100 adults. Each one-category increase in severity was associated with 81% higher adjusted odds (*P* for trend < 0.001).

**Conclusions:**

Long COVID was substantially more prevalent among adults with greater current activity limitation, and the gradient persisted after adjustment for demographic, socioeconomic, and behavioural characteristics. This contemporaneous concentration of post-viral morbidity identifies a marked functional health inequality. Routine surveillance should retain disability-disaggregated measures of post-viral illness, and health systems should ensure that care for persistent post-COVID symptoms reaches people with current activity limitations rather than being confined to dedicated services. Longitudinal studies are needed to establish temporality.

## Background

Long COVID, also termed post-COVID-19 condition, encompasses persistent, recurrent, or newly emerging symptoms after probable or confirmed severe acute respiratory syndrome coronavirus 2 (SARS-CoV-2) infection. The World Health Organisation (WHO) definition generally places symptom onset within 3 months of infection, lasting at least 2 months and unexplained by another diagnosis [1]. Population surveys and clinical studies use broader definitions, commonly with 4–12-week thresholds [2–4]. Modelled global estimates indicate that a substantial minority of people with symptomatic SARS-CoV-2 infection still had persistent fatigue, cognitive, or respiratory symptom clusters 3 months later [5]. Long COVID may affect multiple organ systems, restrict daily activities and participation, and create sustained health, rehabilitation, and social-care needs [2, 3]. As countries have wound down pandemic-specific surveillance and support, long COVID has shifted from an emergency concern to a chronic contributor to population morbidity that must be monitored through routine health-information systems.

Within the International Classification of Functioning, Disability and Health (ICF), activity limitation refers to difficulty executing tasks or actions. Long COVID may produce new, fluctuating, or episodic disability through fatigue, post-exertional symptom exacerbation (PESE), cognitive dysfunction, pain, and breathlessness [2, 6, 7]. Conversely, people with pre-existing disability may carry a greater long-COVID burden because of differences in infection exposure, underlying health, socioeconomic circumstances, and access to appropriate care [8, 9]. Because the present study measures current activity limitation and long COVID concurrently, it cannot distinguish pre-existing disability from limitation caused or worsened by long COVID.

The distribution of this burden is a question of health equity. An estimated 1.3 billion people, about 16% of the world’s population, live with significant disability, and WHO has documented that they die earlier, have poorer health, and are more affected by health emergencies than the general population, largely for avoidable reasons [10]. National evidence suggests marked disability-related inequality in long COVID specifically. In the United States, Hall et al. reported long COVID among 40.6% of people with pre-existing disabilities versus 18.9% of those without [8], and Cohen and van der Meulen Rodgers found associations with both long COVID and related activity limitations [9]. In Scotland, the Coronavirus Infection Survey reported prevalence of 8.6% among people limited a little and 12.0% among those limited a lot by a pre-existing condition, compared with 2.0% among people without such a condition [11].

That survey ended in March 2023, leaving limited nationally representative evidence from the post-emergency phase, after widespread vaccination, repeated population exposure, changing variants, and withdrawal of pandemic-specific surveillance and support. Much existing evidence is descriptive, and whether a severity gradient persists after adjustment for demographic, socioeconomic, and behavioural characteristics remains unclear. Existing estimates are also reported mainly as ratios, whereas absolute differences are what service planners and budget holders need. Using pooled 2023 and 2024 Scottish Health Survey (SHeS) data, we estimated survey-weighted long-COVID prevalence across three levels of activity limitation, tested adjusted associations and a severity trend, and derived adjusted absolute differences. Because the SHeS exposure item closely parallels the Global Activity Limitation Indicator (GALI) used across European health surveys [12], this approach is reproducible in any national survey carrying a comparable functional-limitation question, including in settings, where disability-disaggregated data on post-viral illness are largely absent.

## Methods

### Study design and data source

This was a secondary analysis of the pooled 2023–2024 SHeS combined dataset. SHeS is an annual, nationally representative, repeated cross-sectional survey commissioned by the Scottish Government to monitor health conditions, health-related behaviours, and inequalities in Scotland [13, 14]. The 2023 and 2024 technical reports describe survey methodology and fieldwork [13, 14], whereas the pooled individual-level data used for this analysis were accessed through the UK Data Service data collection [15]. The survey is designed to represent people living in private households; people living in institutions fall outside its sampling frame [14]. In 2023 and 2024, interviews were conducted primarily in participants’ homes using computer-assisted personal interviewing, with a telephone option; different households and individuals were sampled in each year [13, 14].

### Participants

The combined individual-level dataset contained 13,710 respondents interviewed in 2023 or 2024. The present analysis was restricted to adults aged 16 years or older by retaining valid categories of the harmonised adult age-band variable, excluding 4,172 respondents and leaving 9,538 adults. We then excluded 180 adults with missing or non-substantive information for activity limitation or long-COVID status: 176 with a long-COVID response coded as “don’t know” or “item not applicable” and 6 with refused disability information, 2 of whom were in both groups. Finally, 46 adults were excluded because of missing covariate information (sex, *n* = 26; ethnicity, *n* = 8; education, *n* = 17; with some overlap across variables), producing a complete-case analytic sample of 9,312. Overall, 226 adults (2.4% of the eligible adult sample) were excluded. Complete-case analysis was used, and the possibility of selection bias due to missingness is considered in the limitations.

### Exposure: activity-limiting disability

The exposure was the harmonised SHeS measure of activity limitation associated with a long-term condition (LTC), which SHeS defines as a physical or mental health condition or illness lasting, or expected to last, 12 months or more [13]. Respondents reporting a condition were asked whether it limited their activities “a lot,” “a little,” or “not at all” [13]. For the primary analysis, a three-category severity variable was constructed: (1) no activity-limiting LTC, combining respondents without a reported LTC and those reporting a condition that did not limit activities; (2) activities limited a little; and (3) activities limited a lot. Here, “severity” refers specifically to the reported degree of activity limitation across these ordered response categories, not to clinical severity of the underlying condition. Throughout, “activity-limiting disability” refers to this survey-based functional measure, which aligns conceptually with the ICF domain of activity limitation. The item closely parallels the GALI, a single-question measure of participation restriction whose validity and reliability have been reviewed across European populations [12]. It is not equivalent to the Washington Group Short Set, which enumerates difficulty across specific functional domains and is more widely used in censuses and surveys in low- and middle-income countries [16]; estimates from the two instruments are not directly interchangeable.

### Outcome: self-reported long COVID

The outcome was derived from the SHeS long-COVID item. The survey first asked whether respondents had had, or thought they had had, COVID-19, and then defined long COVID as currently experiencing symptoms more than 4 weeks after first having COVID-19 that were not explained by something else [13]. Responses were coded as yes or no; “don’t know” and “item not applicable” responses were treated as missing and excluded. This 4-week survey definition is broader than the WHO clinical case definition [1]; prevalence estimates should, therefore, be interpreted as survey-defined long COVID rather than as prevalence of WHO-defined post-COVID-19 condition.

### Covariates

Covariates were selected a priori on the basis of prior literature, plausible relationships with both current activity limitation and long COVID, and comparable measurement across the two survey years. The adjustment set was intended to assess whether the observed association persisted after accounting for measured demographic, socioeconomic, behavioural, and survey-year differences rather than to estimate a causal effect.

Age was categorised as 16–24, 25–34, 35–44, 45–54, 55–64, 65–74, and 75 years or older. Sex was categorised as male or female. Ethnicity was grouped as White Scottish, White Other British, White Other, Asian, and other minority ethnic. Educational attainment was categorised as degree or higher; Higher National Certificate/Higher National Diploma or equivalent; higher grade or equivalent; standard or other school-level qualification; and no qualifications. Area deprivation was measured using 2020 Scottish Index of Multiple Deprivation (SIMD) quintiles, and cigarette-smoking status as never, former regular, and current smoker. Non-substantive responses for sex, ethnicity, and education were excluded. Survey year was included to account for differences between 2023 and 2024. Individual comorbidities were not included in the adjustment set, because the exposure itself was defined by activity limitation associated with an LTC; residual differences in underlying health status, therefore, remain possible. Vaccination status, number of prior infections, and acute COVID-19 severity were not available in the pooled analytic dataset.

### Statistical analysis

All analyses incorporated the SHeS combined individual calibration weight (int2324wt), strata, and primary sampling unit (PSU) variables [15]. Because PSU and stratum identifiers could recur across annual survey files, year-specific design identifiers were generated by grouping survey year with the original PSU and stratum codes. The final design contained 28 strata and 667 PSUs, none with a single PSU.

Participant characteristics were summarised as unweighted counts and survey-weighted column percentages across the three activity-limitation groups, and differences were evaluated using design-adjusted Pearson tests. Overall and group-specific prevalence of long COVID was estimated with 95% confidence intervals (CIs).

Survey-weighted logistic regression estimated odds ratios (ORs) and 95% CIs for long COVID. Four prespecified models were fitted: an unadjusted model containing activity-limitation severity only; model 1, adjusted for age, sex, and survey year; model 2, additionally adjusted for ethnicity, education, and area deprivation; and model 3, additionally adjusted for cigarette-smoking status. Multicollinearity among covariates in the fully adjusted model was assessed using variance inflation factors (VIFs).

Average marginal predictions from model 3 were used to estimate fully adjusted probabilities of long COVID for each activity-limitation group, and pairwise differences in adjusted probability; these absolute measures were prespecified, because they are more directly interpretable for service planning than ORs. A separate fully adjusted model treated the exposure as an ordinal score to test for linear trend; the categorical model remained primary, because it does not assume equal spacing between severity levels. Additional analyses included a sensitivity analysis that separated the primary reference category into participants with no LTC and those with an LTC that did not limit activities, and an interaction between activity-limitation severity and survey year to test whether the association differed between 2023 and 2024.

Analyses were conducted using StataNow 19.5 (StataCorp, College Station, TX, USA). Tests were two-sided, and *P* < 0.05 was considered statistically significant. The three prespecified pairwise marginal contrasts were evaluated without multiplicity adjustment, because the number of hypothesis-driven comparisons was small; 95% CIs and *p* values are reported. Reporting followed the Strengthening the Reporting of Observational Studies in Epidemiology (STROBE) guidance [17, 18].

## Results

### Sample characteristics

The final analytic sample included 9,312 adults: 5,459 (58.6%) had no activity-limiting LTC, 1,820 (19.5%) reported that activities were limited a little, and 2,033 (21.8%) reported that activities were limited a lot; 4,833 were interviewed in 2023 and 4,479 in 2024. Activity-limitation severity differed across most sociodemographic characteristics (Table 1). Compared with adults without an activity-limiting condition, adults limited a lot were older, more often female, less likely to hold a degree, and more likely to live in the most deprived SIMD quintile. Current cigarette smoking also became more common as severity increased. Survey year did not differ significantly across groups (*P* = 0.105).

**Table 1:** Characteristics of participants by severity of activity limitation

Characteristic	Overall (*N* = 9,312)	No activity-limiting LTC (*n* = 5,459)	Activities limited a little (*n* = 1,820)	Activities limited a lot (*n *= 2,033)	*P*
Age group, years					< 0.001
16–24	655 (11.68)	480 (14.21)	92 (8.08)	83 (7.11)	
25–34	1,164 (15.33)	820 (17.65)	199 (14.30)	145 (9.13)	
35–44	1,244 (15.33)	875 (17.51)	185 (12.37)	184 (11.31)	
45–54	1,264 (16.08)	789 (16.33)	230 (15.60)	245 (15.75)	
55–64	1,734 (17.67)	986 (16.50)	301 (16.86)	447 (21.99)	
65–74	1,874 (13.40)	973 (11.06)	441 (17.25)	460 (17.17)	
≥75	1,377 (10.49)	536 (6.74)	372 (15.53)	469 (17.54)	
Sex					< 0.001
Male	4,143 (47.83)	2,570 (51.44)	767 (44.32)	806 (39.91)	
Female	5,169 (52.17)	2,889 (48.56)	1,053 (55.68)	1,227 (60.09)	
Ethnic background					< 0.001
White Scottish	6,886 (72.43)	3,915 (69.67)	1,389 (74.76)	1,582 (78.81)	
White Other British	1,441 (13.66)	815 (13.06)	310 (16.30)	316 (13.14)	
White Other	520 (6.76)	363 (8.03)	76 (5.37)	81 (4.12)	
Asian	263 (4.20)	203 (5.31)	26 (2.23)	34 (2.52)	
Other minority ethnic	202 (2.96)	163 (3.93)	19 (1.33)	20 (1.41)	
Highest educational qualification					< 0.001
Degree or higher	3,458 (37.68)	2,300 (42.61)	650 (36.58)	508 (23.52)	
HNC/HND or equivalent	1,132 (12.77)	662 (12.24)	234 (14.38)	236 (12.95)	
Higher grade or equivalent	1,317 (15.12)	829 (16.57)	257 (14.44)	231 (11.28)	
Standard/other school level	2,203 (23.01)	1,179 (20.88)	414 (21.90)	610 (30.53)	
No qualifications	1,202 (11.43)	489 (7.70)	265 (12.70)	448 (21.71)	
SIMD 2020 quintile					< 0.001
1, most deprived	1,270 (18.42)	598 (14.74)	257 (20.75)	415 (27.63)	
2	1,642 (19.37)	868 (17.94)	348 (20.63)	426 (22.65)	
3	2,207 (20.13)	1,264 (19.75)	431 (20.15)	512 (21.26)	
4	2,383 (21.38)	1,473 (23.34)	479 (20.47)	431 (16.21)	
5, least deprived	1,810 (20.70)	1,256 (24.24)	305 (18.00)	249 (12.25)	
Cigarette-smoking status					< 0.001
Never smoker	5,841 (64.95)	3,749 (70.94)	1,102 (61.65)	990 (49.50)	
Former regular smoker	2,286 (21.55)	1,162 (18.73)	483 (23.63)	641 (28.33)	
Current cigarette smoker	1,185 (13.51)	548 (10.33)	235 (14.72)	402 (22.16)	
Survey year					0.105
2023	4,833 (51.85)	2,864 (52.22)	875 (48.67)	1,094 (53.51)	
2024	4,479 (48.15)	2,595 (47.78)	945 (51.33)	939 (46.49)	

Values are unweighted n (survey-weighted column %). Percentages account for the combined individual calibration weight, stratification, and clustering. *p* values are from design-adjusted Pearson tests

*HNC* Higher National Certificate, *HND* Higher National Diploma, *LTC* long-term condition, *SIMD* Scottish Index of Multiple Deprivation

### Prevalence of long COVID

There were 682 respondents with self-reported long COVID and 8,630 without. Survey-weighted prevalence was 7.59% (95% CI 6.91–8.34) overall (Table 2). Prevalence rose progressively across activity-limitation categories: 4.89% (95% CI 4.21–5.67) among adults without an activity-limiting LTC, 9.23% (95% CI 7.70–11.04) among those whose activities were limited a little, and 14.42% (95% CI 12.57–16.49) among those whose activities were limited a lot.

**Table 2:** Prevalence of self-reported long COVID by severity of activity limitation

Activity-limitation status	Total, *N*	No long COVID, *n*	Long COVID, *n*	Survey-weighted prevalence, % (95% CI)
Overall	9,312	8,630	682	7.59 (6.91–8.34)
No activity-limiting LTC	5,459	5,190	269	4.89 (4.21–5.67)
Activities limited a little	1,820	1,665	155	9.23 (7.70–11.04)
Activities limited a lot	2,033	1,775	258	14.42 (12.57–16.49)

Counts are unweighted. Prevalence estimates and 95% CIs are survey-weighted and account for calibration weights, stratification, and clustering

*CI* confidence interval, *LTC* long-term condition

### Association between activity limitation and long COVID

**Fig. 1:**
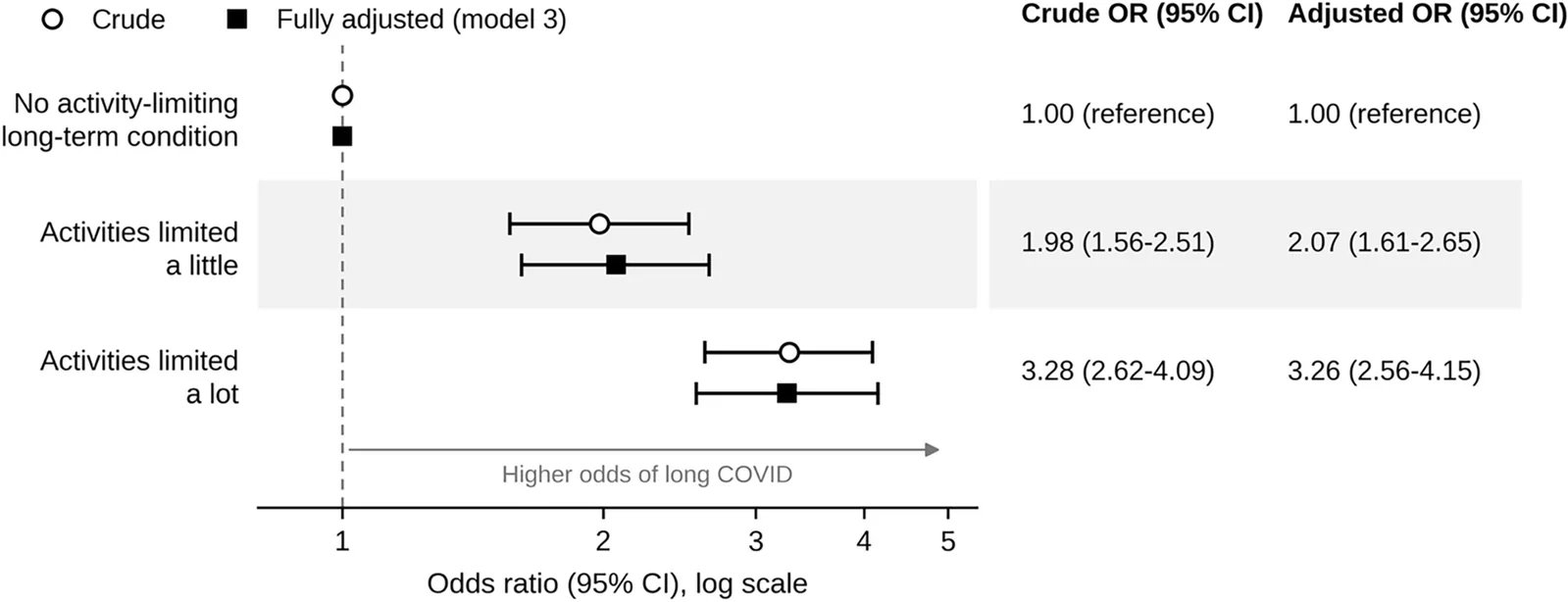
Crude and fully adjusted odds ratios for long COVID by severity of activity limitation. Data are from the Scottish Health Survey 2023–2024 (*N* = 9,312). Open circles denote crude estimates; filled squares denote estimates from model 3, adjusted for age group, sex, survey year, ethnic background, educational attainment, Scottish Index of Multiple Deprivation quintile, and cigarette-smoking status. Horizontal lines are 95% CIs. The dashed vertical line indicates the null value. Adults with no activity-limiting long-term condition are the reference group. The *x*-axis is on a logarithmic scale

In unadjusted survey-weighted logistic regression, compared with adults without an activity-limiting condition, the odds of long COVID were higher among adults whose activities were limited a little (OR 1.98; 95% CI 1.56–2.51) and among those whose activities were limited a lot (OR 3.28; 95% CI 2.62–4.09) (Table 3). Estimates remained stable through sequential adjustment. In the fully adjusted model, the corresponding ORs were 2.07 (95% CI 1.61–2.65) and 3.26 (95% CI 2.56–4.15); both *P* < 0.001 (Fig. 1). Multicollinearity among covariates in the fully adjusted model was assessed using variance inflation factors (VIFs), with no evidence of problematic multicollinearity observed (mean VIF 1.67; maximum VIF 3.32).

**Table 3:** Association between severity of activity limitation and self-reported long COVID

Activity-limitation status	Crude OR (95% CI)	Model 1 OR (95% CI)	Model 2 OR (95% CI)	Model 3 OR (95% CI)	Model 3 adjusted predicted probability, % (95% CI)
No activity-limiting LTC	1.00 (reference)	1.00 (reference)	1.00 (reference)	1.00 (reference)	4.89 (4.16–5.62)
Activities limited a little	1.98 (1.56–2.51)*	2.12 (1.66–2.71)*	2.07 (1.61–2.65)*	2.07 (1.61–2.65)*	9.52 (7.78–11.26)
Activities limited a lot	3.28 (2.62–4.09)*	3.47 (2.75–4.38)*	3.27 (2.56–4.17)*	3.26 (2.56–4.15)*	14.11 (12.10–16.13)
P for linear trend				< 0.001	

Model 1 adjusted for age group, sex, and survey year. Model 2 additionally adjusted for ethnic background, educational attainment, and SIMD quintile. Model 3 additionally adjusted for cigarette-smoking status. Adjusted predicted probabilities are average marginal predictions from model 3. The test for linear trend modelled activity-limitation severity as an ordinal score in the fully adjusted model (OR per one-category increase 1.81; 95% CI 1.61–2.04). All estimates account for survey weights, stratification, and clustering

*CI* confidence interval, *LTC* long-term condition, *OR* odds ratio, *SIMD* Scottish Index of Multiple Deprivation

^*^P < 0.001

Average marginal predictions from the fully adjusted model showed predicted long-COVID probabilities of 4.89% (95% CI 4.16–5.62) among adults without an activity-limiting condition, 9.52% (95% CI 7.78–11.26) among adults limited a little, and 14.11% (95% CI 12.10–16.13) among adults limited a lot. The adjusted absolute difference was 4.62 percentage points (95% CI 2.79–6.46) for limited a little versus no limitation and 9.22 percentage points (95% CI 7.02–11.42) for limited a lot versus no limitation. The difference between limited a lot and limited a little was 4.60 percentage points (95% CI 2.03–7.17); all three prespecified pairwise *p* values were < 0.001. When severity was modelled ordinally, each one-category increase was associated with 81% higher adjusted odds of long COVID (OR 1.81; 95% CI 1.61–2.04; *P* for trend < 0.001).

In the four-category sensitivity analysis, adults with an LTC that did not limit activities had similar odds of long COVID to adults with no LTC (adjusted OR 1.13; 95% CI 0.78–1.62; *P* = 0.519), whereas associations remained elevated among those limited a little (adjusted OR 2.12; 95% CI 1.62–2.76) and limited a lot (adjusted OR 3.34; 95% CI 2.59–4.31), both *P* < 0.001. Adjusted predicted probabilities across the four groups were 4.79%, 5.35%, 9.52%, and 14.13%, respectively. There was no evidence that the association between activity-limitation severity and long COVID differed between 2023 and 2024 (joint *P* for interaction = 0.769).

## Discussion

In this nationally representative pooled analysis of community-dwelling adults in Scotland, greater current activity limitation was strongly associated with a higher prevalence of self-reported long COVID, with a clear severity gradient. Survey-weighted prevalence increased from approximately 5% among adults without an activity-limiting long-term condition to 9% among those limited a little and 14% among those limited a lot. The association persisted after adjustment for demographic characteristics, socioeconomic position, cigarette-smoking status, and survey year, and both the significant linear trend and increasing adjusted probabilities supported a graded relationship. In absolute terms, adults limited a lot had an adjusted long-COVID prevalence approximately 9 percentage points higher than adults without activity limitation—about nine additional cases per 100 adults.

### Comparison with previous evidence

The prevalence gradient closely resembles findings from the Scottish COVID-19 Infection Survey, in which long-COVID prevalence was 8.6% among adults limited a little and 12.0% among adults limited a lot by pre-existing health conditions [11]. The present estimates of 9.23% and 14.42% were somewhat higher, and the overall prevalence of 7.59% exceeded the 3.3% reported for that survey in the 4 weeks ending 5 March 2023 [11]. Direct comparison is inappropriate, because the surveys differed in sampling periods, denominators, question routing, and case definitions. Nevertheless, replication of a severity gradient across independent nationally representative sources strengthens the evidence that long COVID is unequally distributed according to functional health status.

The findings are also consistent with evidence from the United States. Hall and colleagues found markedly higher long-COVID prevalence among people with pre-existing disabilities than among those without [8], and Cohen and van der Meulen Rodgers reported elevated long COVID and long-COVID-related activity limitations in this group [9]. Differences in effect magnitude across studies are expected, because disability definitions, infection denominators, and long-COVID measures vary. The present study adds a contemporary Scottish estimate, distinguishes two levels of limitation severity rather than treating disability dichotomously, and translates adjusted ORs into predicted probabilities and absolute differences that are easier to use in service planning and resource allocation.

### Possible explanations

Several explanations are plausible. People with activity-limiting conditions may have a higher prevalence of chronic illnesses associated with post-COVID sequelae, and systematic review evidence identifies comorbidity, female sex, older age, and severe acute disease as risk factors for post-COVID condition [4]. Disability is also patterned by socioeconomic disadvantage, employment, housing, and access to health services, which may influence infection, acute disease management, recognition of persistent symptoms, and recovery; socioeconomic inequalities in long COVID have been demonstrated in large UK cohorts [19]. Barriers to accessible testing, vaccination, clinical assessment, and follow-up care may further amplify risk or prolong symptoms among disabled people [6, 8, 10].

The age pattern in the adjusted models, with higher odds in middle age and lower odds among the oldest group, is consistent with Scottish surveillance showing the highest prevalence in middle-aged adults [11]. Lower reported prevalence in older adults may reflect differences in survival, symptom attribution, infection history, or participation rather than genuinely lower biological susceptibility. Higher odds among women and the association with deprivation in intermediate models are broadly consistent with the established long-COVID risk factor and inequality literature [4, 19].

### Directionality and the overlap between long COVID and disability

Reverse causation is a central alternative explanation and precludes a temporal interpretation. The SHeS exposure identifies a current condition lasting, or expected to last, at least 12 months that limits activities, and long COVID can itself persist for 12 months and restrict daily activities. Some respondents may, therefore, have been classified as having an activity-limiting condition because of long COVID, or may have experienced worsening of an existing condition after COVID-19. This overlap reflects the close conceptual relationship between long COVID and disability, including its fluctuating and episodic character [6, 7]. The estimates should accordingly be read as the contemporaneous concentration of long COVID among adults with greater activity limitation, not as evidence that disability caused long COVID. For public health practise, the implication is similar under either interpretation: the people most likely to report long COVID are those currently experiencing functional limitation.

### Public health implications

The first implication concerns surveillance. Nearly 1 in 13 adults in Scotland reported long COVID in 2023–2024, several years after the acute phase of the pandemic and after the withdrawal of dedicated infection surveys. General population health surveys have, therefore, become the principal instrument for monitoring post-viral morbidity, and their value depends on retaining both a long-COVID item and a functional-limitation item that can be cross-tabulated. Where surveys drop one or the other, inequalities of the kind reported here become invisible. Disaggregation by disability status is already a routine expectation for monitoring the Sustainable Development Goals and the Convention on the Rights of Persons with Disabilities [10, 16], and post-viral illness should be treated no differently.

The second implication concerns case ascertainment in routine care. Approximately 1 in 7 adults limited a lot reported long COVID, compared with approximately 1 in 20 adults without an activity-limiting condition. Persistent post-COVID symptoms are, therefore, most likely to be encountered among people receiving care for another condition, where fatigue, breathlessness, pain, and cognitive difficulty may be attributed to the pre-existing diagnosis rather than investigated as a possible post-COVID manifestation. Systematically asking about persistent symptoms following COVID-19 across general practise and chronic-disease services, rather than only within dedicated long-COVID clinics, would reduce the risk of this form of diagnostic overshadowing and is achievable without new infrastructure.

The third implication concerns service design. Because long COVID was more prevalent among people with current activity limitations, pathways for post-COVID care should be usable by them: accessible communication, flexible appointment formats, reasonable adjustments, and remote or home-based options where clinically appropriate. Assessment and management also need to accommodate PESE, which is reported by a substantial proportion of people living with long COVID [20]; current guidance supports individualised, symptom-titrated activity and assessment of factors affecting the safety and tolerability of exertion [21], so that pacing and energy conservation are considered rather than fixed exercise progression. Eligibility for support should be determined by current symptoms, function, and need rather than by whether limitation can be shown to have preceded or followed infection, a distinction these data cannot make and one that would exclude people with clinically important overlapping needs [22–24].

The fourth implication concerns generalisability and global evidence gaps. Adults reporting greater activity limitation in this study also differed from those without limitation in educational attainment and area deprivation. Whether similar or larger inequalities occur in settings with weaker primary care and rehabilitation capacity is unknown and requires direct investigation, particularly because disability-disaggregated data on post-viral illness are limited in many settings [10, 16]. The approximately 9-percentage-point adjusted difference observed in Scotland should, therefore, be treated as a context-specific estimate, not as a proxy for the magnitude of inequality elsewhere. Taken together, these findings support retaining functional-limitation measures in routine national surveillance and ensuring that recognition and management of persistent post-COVID symptoms are accessible within general and chronic-disease services. The feasibility and effectiveness of specific service models should be evaluated within each health-system context.

### Strengths and limitations

This study has several strengths, including a large nationally representative sample, use of the full complex survey design, prespecified sequential adjustment, and reporting of absolute as well as relative measures.

Several limitations should also be considered. First, the cross-sectional design precludes establishing temporality and is susceptible to reverse causation. Second, disability was assessed with a broad single-item self-reported functional measure that could not distinguish disability types or identify onset relative to infection; estimates based on the Washington Group Short Set or other multi-domain instruments would not be directly comparable [16]. Third, long COVID was self-reported using a threshold of more than 4 weeks, which is broader than the WHO clinical definition; prevalence should, therefore, be interpreted as survey-defined long COVID rather than directly equated with WHO-defined post-COVID-19 condition. No clinical validation, symptom inventory, date of infection, or measure of PESE or health-service use was available.

Fourth, residual confounding by underlying comorbidity is possible. Individual comorbidities were not included, because they overlap with the exposure definition, and vaccination status, number of prior infections, acute COVID-19 severity, and variant period were not available. Fifth, complete-case analysis excluded 226 adults (2.4% of the eligible adult sample), and selection bias is possible if missingness was related to activity limitation, long COVID, or measured covariates. Sixth, people living in institutions were excluded from the SHeS sampling frame; because institutional residents are disproportionately older and disabled, the findings may understate population inequalities. Seventh, the results describe one high-income national population and should not be extrapolated directly to settings with different infection histories, health systems, or disability profiles. Finally, ORs can exceed prevalence ratios for common outcomes; adjusted predicted probabilities and absolute differences were, therefore, reported alongside ORs to support interpretation on an absolute scale.

## Conclusions

Greater current activity limitation was associated with a substantially higher prevalence of self-reported long COVID among adults in Scotland, with a clear severity gradient that persisted after adjustment for measured demographic, socioeconomic, behavioural, and survey-year differences. This contemporaneous concentration identifies a marked functional health inequality but does not establish whether activity limitation preceded or resulted from long COVID. Routine population health surveys should retain and jointly report long-COVID and functional-limitation measures, so that this inequality remains visible, and services for persistent post-COVID symptoms should be accessible, individualised, and integrated with care for existing conditions rather than confined to dedicated pathways. Longitudinal studies in which disability is measured before SARS-CoV-2 infection are needed to distinguish pre-existing vulnerability from disability caused or worsened by long COVID.

## Data Availability

The data that support the findings of this study are available from the UK Data Service, but restrictions apply: the data were used under licence for the current study and are not publicly available without registration. The Scottish Health Survey 2024, including the 2023–2024 combined datasets, can be obtained by registered users from the UK Data Service (study number 9518) subject to the repository terms of use [15]. The analytic code is available from the corresponding author on reasonable request.

## Abbreviations

CI: Confidence interval
COVID-19: Coronavirus disease 2019
GALI: Global activity-limitation indicator
HNC: Higher National Certificate
HND: Higher National Diploma
ICF: International Classification of Functioning, Disability and Health
LTC: Long-term condition
OR: Odds ratio
PESE: Post-exertional symptom exacerbation
PSU: Primary sampling unit
SARS-CoV-2: Severe acute respiratory syndrome coronavirus 2
SHeS: Scottish Health Survey
SIMD: Scottish Index of Multiple Deprivation
STROBE: Strengthening the Reporting of Observational Studies in Epidemiology
WHO: World Health Organisation
